# Identification of complex regional pain syndrome in the upper limb: Skin temperature asymmetry after cold pressor test

**DOI:** 10.1080/24740527.2018.1504283

**Published:** 2018-08-21

**Authors:** Tara Packham, Joy MacDermid, James Bain, Norm Buckley

**Affiliations:** a Hamilton Health Sciences, Regional Rehabilitation Program, Hamilton, Ontario, Canada; b School of Physical Therapy, Elborn College, Western University, London, Ontario, Canada; c Department of Surgery, Hamilton Health Sciences, Hamilton, Ontario, Canada; d Michael G. DeGroote Institute for Pain Research and Care, Hamilton, Ontario, Canada

**Keywords:** complex regional pain syndrome, sensitivity, specificity, validity, skin temperature asymmetry, cold pressor test

## Abstract

**Background:**

Skin temperature asymmetry (SkTA) may assist in early identification of complex regional pain syndrome (CRPS), but previous work has been limited by methodological shortcomings including failure to account for the cutaneous nerve distribution where temperature is measured and reliance on laboratory equipment not clinically available. Pilot work suggested that a cold pressor test (CPT) provided a consistent thermoregulatory stress and might increase sensitivity/specificity of SkTA measurements generated reliably by handheld infrared (IR) thermometers.

**Aims:**

This study investigated the sensitivity, specificity, and validity of SkTA in the upper limb to identify CRPS.

**Methods:**

This study was part of a larger clinical trial (the SARA study: www.clinicaltrials.gov NCT02070367). Using IR thermometers, we evaluated SkTA over major peripheral nerve distributions in the hands before and after immersing a single foot in 5°C water for 30 s. Participant groups included healthy volunteers, CRPS, known nerve injury, and hand fracture.

**Results:**

SkTA was measured in 65 persons, including 17 persons with CRPS (meeting Budapest criteria). Analysis of variance for *n* = 378 SkTA observations supported diagnosis, CPT, and nerve distribution as significant predictors (*P* < 0.001) explaining 94% of the variance. Post CPT, sensitivity for a >1.5°C SkTA improved to 82.4% from 58.8%, whereas specificity dropped from 56.3% to 43.8%.

**Conclusion:**

This study adds further support for the accuracy of SkTA as a diagnostic indicator of CRPS. Further precision in estimates will be gained from larger studies, which should also seek to replicate our findings for SkTA in the lower limbs.

## RÉSUMÉ

**Contexte**: L’asymétrie de la température cutanée peut contribuer à la détection précoce du syndrome douleureux régional complexe (SDRC), mais les travaux réalisés par le passé se sont caractérisés par des insuffisances méthodologiques, parmi lesquelles l’omission de tenir compte de la distribution des nerfs cutanés à l’endroit où la température était mesurée et l’utilisation d’équipement de laboratoire non accessible cliniquement. Selon un projet pilote, un test au froid donne lieu à une tension uniforme dans la thermorégulation et pourrait donc acccroître la sensibilité/spécificité des mesures de l’asymétrie de la température cutanée obtenues de manière fiable par des thermomères infrarouges à main.

**But**: Cette étude portait sur la sensibilité, la spécificité et la validité de l’asymétrie de la température cutanée dans les membres supérieurs pour détecter le SDRC.

**Méthodes**: Cette étude faisait partie d’un essai clinique plus vaste (l’étude SARA : www.clinicaltrials.gov NCT02070367). À l’aide de thermomètres infrarouges, nous avons mesuré l’asymétrie de la température cutanée sur d’importantes distributions de nerfs périphériques dans les mains avant et après l’immersion d’un pied dans l’eau à 5^o^ C pendant 30 secondes. Les groupes de participants étaient les suivants : volontaires en bonne santé, SDRC, lésion nerveuse connue et fracture à la main.

**Résultats**: L’asymétrie de la température cutanée a été mesurée chez 65 personnes, dont 17 personnes souffrant de SDRC (répondant aux critères de Budapest). L’analyse de la variance des observations d’asymétrie de la température cutanée pour n = 378 a démontré que le diagnostic, le test au froid et la distribution des nerfs étaient des prédicteurs significatifs (p < 0,001) expliquant 94 % de la variance. La sensibilité après le test au froid pour une asymétrie de la température cutanée  > 1,5 ° C avait augmenté de 58,8 % à 82,4 %, tandis que la spécificité avait diminué de 56,3 % à 43,8 %.

**Conclusion**: Cette étude vient appuyer l’exactitude de l’asymétrie de la température cutanée en tant qu’indicateur d’un diagnostic de SDRC. La réalisation d’études de plus grande envergure permettra d’en arriver à des estimations plus précises. De telles études devraient également chercher à reproduire nos résultats relatifs à l’asymétrie de la température cutanée dans les membres inférieurs.

## Introduction

Complex regional pain syndrome (CRPS) is a painful disorder that may develop after minor trauma but with the potential for chronic and significant disability.^1,2^ CRPS is commonly divided into two categories (type I and type II), distinguished only by the presence of a known nerve injury in type II, replacing earlier nomenclature of reflex sympathetic dystrophy and causalgia.^3^ It is estimated to affect as many as 30%–40% of patients following upper extremity injuries or surgery,^4,5^ occurs in three times more women than men, and is seen more often in the upper than lower extremities.^6^ The disparate symptoms of CRPS are notoriously variable both between and within individuals, making it difficult to diagnose.^7^ Accordingly, there is currently no accepted diagnostic test for CRPS,^8^ and diagnosis is made using validated criteria based on both patient report and clinician observation.^9,10^

Measuring skin temperature asymmetry between limbs has been proposed to assist in the diagnosis of CRPS^11^ and peripheral nerve injuries (PNIs),^12^ but most studies have used costly lab-based equipment not available in clinical practice settings.^13–15^ Mechanisms underlying the vasomotor changes seen are not clear, and both afferent and efferent pathways have been proposed.^16^ However, the focus of sympathetic stress testing has often been to test the vasomotor response as a surrogate for function of the sympathetic nervous system^17,18^ and not purely as a diagnostic indicator for a pain syndrome that may include multiple peripheral and cortical mechanisms.^19^ Skin temperature evaluations for this purpose are often a secondary test to complement Doppler measures of blood flow. Several studies suggest the sensitivity of temperature asymmetry for diagnosis of CRPS might be improved if measured during sympathetic stress produced by exposing the patient to some form of temperature extreme^20–22^ or by tracking temperature over a longer time period to see the variations elicited by daily stressors.^23^

Pilot work demonstrated the reliability of skin temperature measurements using inexpensive handheld infrared (IR) thermometers for temperature comparisons between limbs and established the safety of a cold pressor test (CPT) for stressing the thermoregulatory system in the clinical setting.^24^ However, given that CRPS II (1) by definition includes an element of PNI and (2) is often related to trauma, it follows that any validation of skin temperature asymmetry for the identification of CRPS must also address the potential for temperature differences in nerve injuries^12,25^ and posttraumatic inflammation.^26,27^ Further, it has been demonstrated that small fiber neuropathy may be present in CRPS I,^28^ which illustrates the need to investigate whether the temperature asymmetry seen in nerve injuries differs from the presentation in CRPS.

The purpose of this study is to explore the sensitivity and specificity of skin temperature asymmetry before and after a CPT for the identification of CRPS and to test hypotheses about temperature asymmetry that would support the validity of this form of evaluation. Therefore, our primary research question is the following: Will temperature asymmetry between limbs after a CPT be more sensitive and specific for the diagnosis of CRPS than temperature differences measured without cold stress? Secondary questions include the following: (1) Do persons with CRPS demonstrate more skin temperature asymmetry between affected and unaffected limbs after a CPT when compared to persons with PNIs, recent hand trauma, or healthy normals? and (2) What is the most sensitive and specific cut-point for temperature asymmetry in CRPS?

## Materials and methods

### Design and setting

This prospective study was conducted at the outpatient Hand Therapy Clinic at a regional trauma center and teaching hospital in Hamilton, Ontario; data were collected between September 2014 and September 2016. Baseline evaluations were conducted by one of two independent assessors with expertise in hand rehabilitation: one physiotherapist and one occupational therapist. All participants gave written informed consent, and the study was approved by the local ethics committee (Hamilton Integrated Research Ethics Board).

### Subjects

As part of a larger study (the SARA study: www.clinicaltrials.gov NCT02070367), patient participants were recruited from local hand therapy facilities and pain programs. Inclusion criteria for patients were (1) a diagnosis of CRPS meeting the Budapest clinical criteria^29^ in a single upper limb or (2) a unilateral PNI in the upper limb verified intra-operatively or (3) a recent hand fracture (within 8–12 weeks of fracture). All were confirmed by medical record to ensure eligibility. Healthy volunteers for temperature testing were also recruited from staff and learners at the trauma center. Additional inclusion criteria for all participants were persons over age 16 and able to provide informed consent. Exclusion criteria for all participants were (1) history of cardiac or vascular disease, known sensitivity to sympathetic stress, cold intolerance, or medically unstable; (2) presence of confounding diagnoses: whiplash, nerve root compression, metabolic diseases such as diabetes or thyroid problems, and/or peripheral neuropathy; (3) open wounds on any of the testing sites; (4) participant has previously been treated with implanted spinal cord stimulator; or (5) participant is currently receiving injections of pain medications into the affected region (i.e., lidocaine infusion, botulinum toxin, stellate ganglion blockade).

Target sample size for the explorations of sensitivity and specificity was based on variability estimates from our pilot work comparing person with CRPS with healthy volunteers,^24^ indicating that the false positive rate among healthy controls is 0.1. Assuming that the true positive rate for persons with CRPS is 0.4, we estimated that we would need to study 17 experimental subjects and 17 control subjects to be able to reject the null hypothesis that the rates of temperature asymmetry of 1.2°C or greater for both experimental and control subjects are equal with a probability (power) of 0.84.^30^

### Study measures

**Figure 1.**
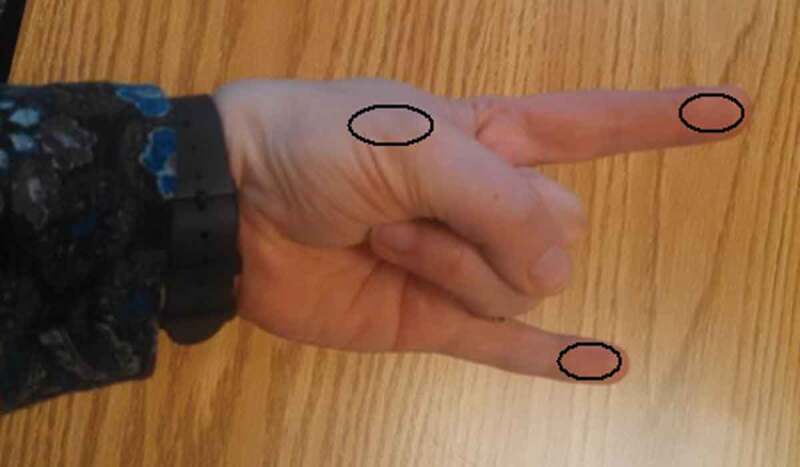
Testing areas for each of the major peripheral nerve distributions in the hand.

Demographic and clinical information was collected from patient participants regarding age, sex, diagnosis, time since injury, dominance, affected side, and screening for inclusion/exclusion criteria. Measures of skin temperature symmetry utilized the safe and reliable equipment and procedures previously described in our pilot work.^24^ Participants were asked to sit in a climate-controlled room for 20 min prior to temperature measurements and were asked to wipe their hands with a dry towel to remove any moisture. The cutaneous territory for each of the major peripheral nerve branches in the hand (see Figure 1) was scanned using an IR thermometer (model TH03F; Radiant Innovation, Inc., Taiwan; www.radiantek.com.tw) for 3 s and the temperature was recorded for both hands. Participants were then asked to remove a sock and/or shoe from one foot and immerse the foot in an insulated vessel of 5°C water for 30 s (CPT). Immediately after withdrawing the foot from the cold water, the rater repeated the temperature readings.

As part of the larger study, patient participants also completed self-report questionnaires about pain and disability and were evaluated for impairments in movement, power, and sensation; these results will be reported elsewhere.

### Statistical analysis

After importing into Stata13 software, means and frequencies were generated for all variables and plotted to examine for potential data entry errors. Temperature measures of the affected and unaffected hands for patients and right and left hands for healthy volunteers for the same occasion (pre and post CPT) and same nerve distribution were used to calculate skin temperature asymmetry as both the difference from right to left (indicating the direction of the difference relative to the right hand temperature) and an absolute difference value. We chose to use the right hand as our reference value because this was both relevant for healthy volunteers and acted as a surrogate for affected and unaffected side, because there was not a consistent presentation of colder or warmer affected limbs. Based on our pilot work,^24^ we predicted that there would not be a consistent response in the direction of temperature change in the affected hand after the CPT in the CRPS group.

Overall sensitivity and specificity values were generated using an online calculator (https://www.medcalc.org/calc/diagnostic_test.php) based on 2 × 2 tables for various cut-points of temperature differences, comparing persons with CRPS vs. all other groups combined and evaluating values from both before and after the CPT. For the primary analysis, positive cases were defined by at least one positive test of skin temperature asymmetry (SkTA; at the level of the associated cut-point) in *any* of the three nerve distributions and therefore were considered negative cases if there was no asymmetry in any of the triad of temperature measures. We also created receiver operating characteristic (ROC) curves^31^ using the largest temperature difference measured in any of the three nerve distributions both pre cold pressor testing and post cold pressor testing and calculated the area under the curve. Analysis of variance was conducted to consider the contributions of (1) diagnostic group, (2) occasion (pre/post CPT), and (3) cutaneous area/peripheral nerve distribution measured to the observed skin temperature asymmetry (using nested variables to account for the inherent correlations of multiple measures taken from the same subject). Separate analyses of healthy volunteers and patients were conducted initially to verify that the contributions of occasion and cutaneous area were present even in the healthy controls.

## Results

### Participants

Skin temperature asymmetry was measured in 65 persons, including 17 persons meeting the Budapest clinical criteria for CRPS. Patient participants also included persons with nerve injuries of the upper limb or who had sustained a hand fracture within the past 8 weeks. Complete demographic and clinical information is summarized in Table 1 (patients) and Table 2 (healthy volunteers).

**Table 1.** Patient demographics and baseline characteristics (*N* = 33).

Characteristics		Mean	SD	Range
Age		46.2	13.7	15–76
Duration of injury or pain (months)		22.4	38.5	1–184
SkTA pre CPT (°C)	Median	0.99	1.22	0.1–6.4
	Ulnar	1.09	0.85	0–3.6
	Radial	0.81	0.68	0–2.7
SkTA post CPT (°C)	Median	1.43	1.31	0.1–5.3
	Ulnar	1.13	1.00	0–4.9
	Radial	1.01	0.95	0–3.4
Characteristic		Frequency	Percentage	
Gender		M = 16 F = 17	M = 48.5% F = 51.5%	
Diagnosis		CRPS = 17 PNI = 10 Fracture = 6	51.5% 30.3% 18.2%	
Dominance		R = 21 L = 12	R = 63.6% L = 36.4%	
Side of injury		R = 17 L = 16	R = 51.5% L = 48.5%	

SkTA = skin temperature asymmetry; CPT = cold pressor test; M = male; F = female; R = right; L = left; CRPS = complex regional pain syndrome; PNI = peripheral nerve injury.

**Table 2.** Healthy volunteers (*n* = 32).

Characteristics		Mean	SD	Range
Age		39.3	12.1	20– 59
SkTA pre CPT (°C)	Median	0.40	0.39	0– 1.4
	Ulnar	0.62	0.46	0–2.1
	Radial	1.67	1.38	0–5.0
SkTA post CPT (°C)	Median	0.37	0.36	0–1.4
	Ulnar	0.63	0.50	0–2.0
	Radial	0.26	0.21	0– 0.9
Characteristic		Frequency	Percentage	
Gender		M = 9 F = 23	M = 32.1% F = 71.9%	
Dominance		R = 29 L = 3	R = 90.6% L = 9.3%	

SkTA = skin temperature asymmetry; CPT = cold pressor test; M = male; F = female; R = right; L = left.

### Temperature data

In healthy volunteers at baseline and post CPT, skin surface temperatures ranged from 21.8°C to 35.0°C (Table 3). Mean absolute skin temperature asymmetry in all nerve distributions ranged from 0.37°C to 1.67°C; however, the range of absolute differences was 0°C to 5.0°C (*n* = 168 paired measures, including both pre and post measures). It is worth noting that extreme values were recorded in several cases in the radial nerve distribution (Table 2). In patients, skin temperature measures ranged from 21.8°C to 36.1°C across occasions (Table 3). Mean absolute skin temperature asymmetry in all nerve distributions was 0.81°C to 1.43°C (see Table 1), but the range of absolute differences was 0°C to 6.4°C (*n* = 198 paired measures, including both pre and post measures).

**Table 3.** Temperature values by nerve distribution.

Patient characteristics		Mean	SD	Range
Median	Pre CPT	A = 31.9	1.7	27.0–34.4
		U = 31.6	1.7	27.1–34.1
	Post CPT	A = 31.8	1.7	27.0–34.1
		U = 31.5	1.7	27.0–33.7
Ulnar	Pre CPT	A = 31.9	1.8	28.5–34.9
		U = 31.8	1.8	27.3–34.7
	Post CPT	A = 31.5	1.8	28.0–34.8
		U = 31.5	2.1	27.1–34.9
Radial	Pre CPT	A = 31.8	2.7	25.2–36.1
		U = 32.0	2.2	27.5–36.0
	Post CPT	A = 31.2	2.8	24.7–35.6
		U = 31.5	2.3	26.3–35.2
Healthy volunteer characteristics		Mean	SD	Range
Median	Pre CPT	R = 28.8	3.8	21.8–35.0
		L = 29.0	3.6	23.2–34.9
	Post CPT	R = 28.5	3.8	22.7–34.8
		L = 28.7	3.6	23.2–35.0
Ulnar	Pre CPT	R = 30.4	2.5	24.5–34.4
		L = 30.8	2.3	26.2–34.5
	Post CPT	R = 30.5	2.2	27.0–34.3
		L = 30.9	2.0	27.2–34.4
Radial	Pre CPT	R = 31.0	1.5	28.2–33.4
		L = 31.0	1.4	28.1–33.7
	Post CPT	R = 31.0	1.6	27.7–33.4
		L = 31.0	1.5	28.6–33.5

CPT = cold pressor test; A = affected hand; U = unaffected hand; R = right hand; L = left hand.

### Diagnostic accuracy

The best combination of sensitivity and specificity at rest (prior to use of the cold pressor) was seen for (1) sensitivity at >1.0°C SkTA = 88.2% and (2) specificity of 81.3% at a cut-point of >2.0°C. However, sensitivity dropped to 56.3% post cold pressor at this cut-point. The CPT did appear to enhance the sensitivity at a cut-point above 1.0°C (see Table 4); nonetheless, the associated confidence intervals for estimates at all cut-points were substantial, reflecting the small sample size.

**Table 4.** Sensitivity and specificity estimates.

Diagnostic value	Cut-point	Pre CPT (95% CI)	Post CPT (95% CI)
Sensitivity	>1.0°C SkTA	88.2 (63.6–98.5)	88.2 (63.6–98.5)
Specificity		37.5 (15.2–64.6)	18.8 (4.1–47.5)
Sensitivity	>1.5°C SkTA	58.8 (32.9–81.6)	82.4 (56.6–96.2)
Specificity		56.3 (29.9–80.3)	43.8 (19.8–70.1)
Sensitivity	>2.0°C SkTA	29.4 (10.3–56.0)	52.9 (27.8–77.0)
Specificity		81.3 (54.4–96.0)	56.3 (29.9–80.3)

CPT = cold pressor test; SkTA = skin temperature asymmetry.

**Figure 2.**
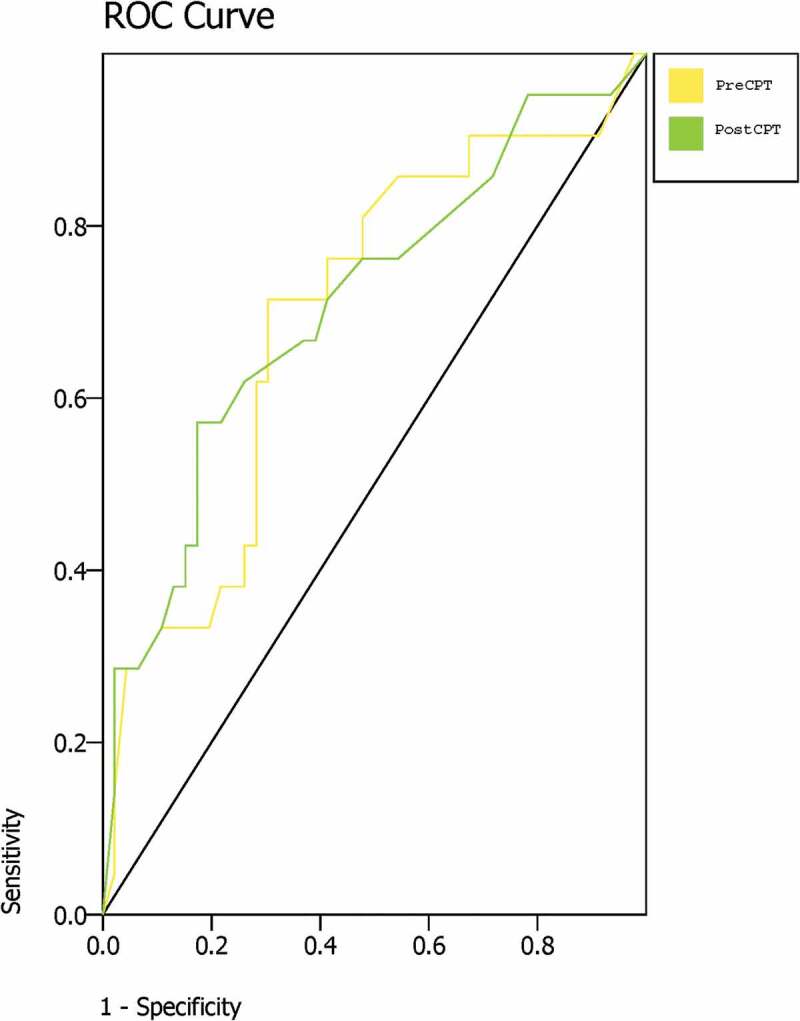
ROC curve plotted using the largest asymmetry value from each subject pre and post CPT.

ROC curves using the largest difference (asymmetry) value from any of the three nerve distributions measured were constructed using both the pre–cold pressor and post–cold pressor values (Figure 2). For temperature asymmetry at baseline, the AUC was 0.70 (95% confidence interval [CI], 0.59–0.82), *P* = 0.008. For temperature asymmetry after cold pressor testing, the AUC was 0.71 (95% CI, 0.60–0.83), *P* = 0.005.

Analysis of variance (ANOVA) of temperature differences in healthy volunteers by occasion (pre or post cold pressor) and by nerve distribution (accounting for inherent associations from multiple test sites within each participant) was statistically significant, F(_168,108_) = 5.99, *P* < 0.001, accounting for 92% of the variance seen. Post hoc contrasts demonstrated that the degree of temperature differences (asymmetry) differed significantly across nerve distributions (*P* < 0.001), accounting for multiple nerves being tested in each participant. ANOVA of temperature differences in patients by occasion, nerve distribution, and diagnostic group (CRPS, fracture, or nerve injury) with nested variables to account for multiple test sites in the same participant and considering potential interactions was highly statistically significant, F(_186,123_) = 7.99, *P* < 0.001, explaining 94% of the variance.

Combining both data sets, ANOVA yielded F(_378, 251_) = 7.53, *P* < 0.001, with diagnostic group categorized as CRPS or not CRPS (including healthy volunteers), again accounting for nerve and occasion being nested within the individual cases. This also explained 94% of the variance in skin temperature asymmetry.

## Discussion

### Sensitivity and specificity

We studied the potential of a CPT to increase the sensitivity and specificity of SkTA for the identification of complex regional pain syndrome. We hypothesized that temperature asymmetry could help distinguish from other forms of limb trauma and sought to identify the most useful cut-point for SkTA in CRPS. Niehof and colleagues measured SkTA with IR thermometers in the center of the hands or feet in both volar and dorsal planes and reported limited validity and utility for differentiation of CRPS from other fracture sequelae, with a maximum sensitivity of 71% and specificity of 64% at a cutpoint of 1.0°C.^32^ Wasner et al.^11^ compared the diagnostic value of SkTA in CRPS I, finding a sensitivity of 32% at a cut-point of 2.0°C with static testing, which increased to 76% during dynamic sympathetic activation using thermal suits. They reported 100% specificity at rest and 93% after dynamic testing. We found the best sensitivity (88.2%) at a cut-point of 1.0°C and specificity (81.3%) at a cut-point of 2.0°C when comparing to controls (healthy volunteers or patients with either nerve injury or fracture). Using ROC curve analysis, we found highly similar areas under the curve of 0.70 and 0.71 for SkTA before and after cold pressor testing, respectively. Repeating our methods with larger samples is warranted to see whether the results can provide stronger support for one testing method over the other.

### Cold pressor testing

Wasner tested bilateral cutaneous perfusion with laser Doppler after activation of the sympathetic vasoconstrictor pathway with cold stress and noted an increased phasic reflex (increasing constriction) on the affected side in a case of acute CRPS I but reported no differences in reflex activity in the same patient 7 weeks later or in two healthy volunteers.^17^ Though he also measured skin temperature, these measures were only taken during testing of the tonic reflex with whole-body temperature manipulation. Birklein and colleagues tested vasoconstriction responses in 14 persons with CRPS I after CPT^18^ and failed to find significant differences in perfusion compared to control subjects. However, skin temperature was only measured in central points on the hand after the cold exposure.^18^ One possible explanation is that cold pressor testing elicits less variation in proximal areas of the limbs than in the distal finger pads.^33^ Of note, visual examination of the data in the figure^18^ suggests that some subjects with CRPS I experienced increased perfusion, whereas others experienced decreased perfusion after cold pressor testing, supporting change in symmetry as the relevant variable rather than the direction of the change. We made similar observations of bidirectional change in our pilot work,^34^ which informed our decision to focus on symmetry and absolute temperature change after cold pressor testing.

Krumova et al. posited that taking measurements over a longer interval of time instead of at a single time point would address the within-subject variability and increase the validity of SkTA measures.^23^ They compared dynamic temperatures in persons with CRPS, persons with limb pain of another origin, and healthy volunteers by taking readings every minute across a period of at least 5 h while the person completed daily activities. Though both patient groups differed from healthy volunteers, a composite score accounting for temperature oscillations, time with temperature asymmetry, and asynchronicity (when one hand increased in temperature simultaneous with the opposite hand decreasing in temperature) was reported to yield the highest sensitivity and specificity for the identification of CRPS at specificity = 67% vs. non-CRPS and 79% vs. healthy controls and sensitivity = 73% compared to other patients and 94% in comparison to healthy controls.^23^ We sought to address variability more simply by using a CPT to create a standardized stress to the thermoregulatory system, hypothesizing that this would improve the sensitivity of SkTA. Sensitivity was increased at higher cutoff values (1.5°C or 2°C) but was unchanged at 1.0°C (see Table 3). However, dynamic testing using a cold pressor did not enhance specificity. In clinical practice, this suggests that an SkTA of 1°C in any of the three major nerve distributions of the hand will identify most cases of upper limb CRPS and a 2°C difference at baseline will rule out most other conditions. However, it is important to note that the substantial confidence intervals (see Table 3) reflect our small sample size and the values seen here may not generalize to larger samples. The largest study of SkTA to date used thermography to examine SkTA under resting conditions in a large cohort (*n* = 296) of persons with CRPS I or CRPS II^35^ and reported that the mean temperature difference between affected and unaffected limbs was −0.72°C ± 1.65°C. They reported that 44.3% of their sample did not have a skin temperature asymmetry of greater than 1°C and concluded that symptom chronicity was not an important predictor of SkTA.^35^ However, it is also worth noting that their population contained more males and more persons with lower limb CRPS than upper limb CRPS and therefore may not be representative of the broader CRPS population.

Cooke et al.^22^ studied responses to mild cold stress in the symptomatic (ipsilateral) hand on mean hand temperature measured using IR thermography in 20 persons with CRPS I, also tracking rewarming and then repeating cold stress on the unaffected (contralateral) hand. Some participants saw rewarming temperatures increase from baseline, whereas others failed to return to baseline; these responses also varied between cold stress to the ipsilateral or contralateral hand.^22^ At baseline, they reported a mean temperature asymmetry of 0.3°C in healthy controls and mean SkTA of 0.6°C in participants with CRPS I. We also observed a variety of responses in our patient groups after exposure to a strong cold stress on the foot: though skin surface temperatures generally decreased in both hands in response to cold (suggesting vasoconstriction), the affected hand would drop to a greater extent. However, we did have several cases where the affected hand increased in temperature after cold stress and asymmetry disappeared. Given the heterogeneity of responses seen relative to cold pressor testing,^18,22,24^ we posit that this may represent elements of fear or threat anticipation that modulate the peripheral vasomotor responses measured relative to activity and environmental stimuli.^7,23^

Baron and Maier^36^ compared the symmetry of sympathetic vasoconstriction responses in persons with CRPS I and concluded that dynamic testing of SkTA was superior to testing of static differences. Birklein et al.^18^ compared the sympathetic vasoconstriction responses to several different forms of stressors, including a CPT, in 20 persons with relatively acute CRPS (mean duration 8.5 weeks, range 2–70). For cold pressor testing, the opposite set of limbs (hands vs. feet) was immersed in ice water for 1 min. Though they reported a drop in laser Doppler blood flow measures on the pad of the fifth digit during the cold exposure, skin temperature measures in the central area of the dorsum of the painful extremity did not show statistically significant SkTA when measured immediately after the vasoconstriction testing. However, our earlier pilot work^24^ and results reported here suggest that this central measurement point may have failed to capture the distinct differences in SkTA between peripheral nerve distributions. We found that SkTA differed across nerve distributions even in healthy volunteers (*P* < 0.001), and this finding was replicated in patients (*P* < 0.001). This is concordant with normal values for thermography findings reported by Uematsu et al.^37^—they reported mean SkTA for 40 different regions of interest across the entire body and noted that mean differences of the index finger (median nerve) were 0.52°C ± 0.46°C, mean differences of the small finger (ulnar nerve) were 0.45°C ± 0.39°C, and the dorsum of the hand (combined median, ulnar, and radial) measured 0.31°C ± 0.25°C.^37^ Our findings in healthy volunteers using skin surface IR thermometers were similar, with mean differences measured at the tip of the index (median nerve) of 0.37°C ± 0.36°C and mean differences at the base of the small finger (ulnar nerve) of 0.63°C ± 0.50°C.

### Identification of CRPS vs. differential diagnosis

Though findings of difference in skin temperature asymmetries between healthy volunteers and persons with CRPS are necessary to validate the underlying concept, the clinical challenge is differential diagnosis: to distinguish between persons with CRPS and those with other conditions. In an experimental animal model of nerve injury, mean SkTA of 1.0°C–2.4°C was reported in the ulnar nerve distribution.^12^ Although they found significant differences between SkTA in healthy volunteers and CRPSI after cold exposure, Cooke et al. failed to distinguish between persons with CRPSI and patients with nociplastic pain. Birklein et al.^38^ reported statistically significant temperature increases in the affected limb of both fracture patients (*n* = 22) and persons with CRPS (*n* = 24) but were unable to distinguish between the groups on the basis of static SkTA. Wasner and colleagues did find statistically significant differences between persons with CRPS, persons with limb pain of other origin, and healthy controls in peak absolute SkTA during whole-body cooling^39^ but, given the technical demands of their materials and methods, this is impractical to inform clinical decision making. We sought to validate this measurement method by collecting SkTA readings in persons with CRPS, PNI, or recent fractures and healthy volunteers both in static conditions (pre-CPT) and after an indirect thermoregulatory stress intended to create a standardized dynamic measurement condition (post-CPT). Further, we took measures in each of the three major peripheral nerve distributions of the upper limb and combined this data to test whether more SkTA was seen in persons with CRPS. Using robust statistical analyses with nested variables, we were able to explain 94% of the variance and demonstrate statistically significant differences in SkTA between patient groups, while accounting for different cutaneous nerve distributions and static or dynamic testing methods.

### Strengths and limitations

There are several limitations to this work. We only studied SkTA in the upper extremity, and it is not clear whether there would be differences in SkTA found in the lower limbs, because this question has not been addressed by other authors employing mixed samples.^35,38^ Our sample included a spectrum of symptom presentation and duration; however, others have suggested that time course was not a significant predictor of SkTA^35^; future work should consider whether there are important patterns of symptoms (such as vasomotor changes) that influence or predict skin temperature asymmetry after cold pressor testing. We also did not distinguish between warm and cool subtypes of CRPS,^1^ and it is unknown whether the baseline level of vasoconstriction or vasodilation would be an important influence on SkTA. Further, we chose to use absolute values for temperature change and did not address the direction of the temperature change seen after cold pressor testing. In combination, this may have missed an opportunity to identify phenotypes of vasomotor instability with unique responses that could explain why some individuals saw a decrease in SkTA after cold pressor testing as a cold hand became warmer or a warm hand became cooler.

Our samples were small, and underpowering of sensitivity and specificity estimates is illustrated by the confidence intervals reported. We did not distinguish between CRPS I and II because we anticipated that our sample would be too small for meaningful subgroup analysis. Though our methods still employed multiple measurements (nerve distributions, and pre/post CPT), they are both quicker and more practical than the sum score proposed by Krumova^23^ and use standardized methods and inexpensive materials to produce similar findings to those generated in detailed laboratory testing.^11^ However, we did not independently calibrate the IR thermometers, which may have introduced systematic measurement error. Finally, we investigated sensitivity and specificity at preselected thresholds of 1.0°C, 1.5°C, and 2.0°C rather than exploring the ideal value to maximize diagnostic precision using ROC curves. Given the small sample size and resultant likelihood that this value would change with retesting, we thought it more helpful to compare to thresholds previously used in the literature at this early stage of investigation.

### Clinical application

Though this body of evidence is not sufficiently robust to proclaim the evaluation of SkTA as a diagnostic standard for the identification of CRPS, we feel that this investigation adds to the literature on how and why health professionals might measure skin temperature to inform their clinical diagnosis of CRPS. Clinicians seeking to robustly evaluate SkTA should (1) ensure environmental acclimatization for at least 10 min prior to taking any temperature measures,^22,37^ (2) gently wipe or blot the area to be measured with a towel to remove moisture,^37^ (3) support the limb without contact on the area to be measured while ensuring that the limbs do not cross midline,^40^ (4) measure anatomically matched cutaneous nerve territories of glabrous skin,^24^ (5) use measurement equipment with a temperature range and sensor size appropriate for the target skin areas,^41^ and (6) take measures under both static and dynamic conditions of rest and thermoregulatory challenge.^18,36^ Cold stress appears to increase the sensitivity but not specificity of SkTA measures; accordingly, the risk vs. reward appraisal should consider the potential discomfort of the patient during this procedure weighed against the value of the information generated. Though more research is needed to generate stable estimates of sensitivity and specificity, we advocate for a continued focus on simple equipment that can be easily replicated in the clinical setting.
